# Supplementary material for: A Binary Mixture of Emamectin Benzoate and Chlorantraniliprole Supplemented with an Adjuvant Effectively Controls Spodoptera frugiperda
Source: Insects. 2022 Dec 15;13(12):1157. doi: 10.3390/insects13121157 (PMC9785781; doi:10.3390/insects13121157)
Supplement: Supplementary file 1 [file insects-13-01157-s001.zip › insects-2073334-supplementary.pdf]

Supplementary figures

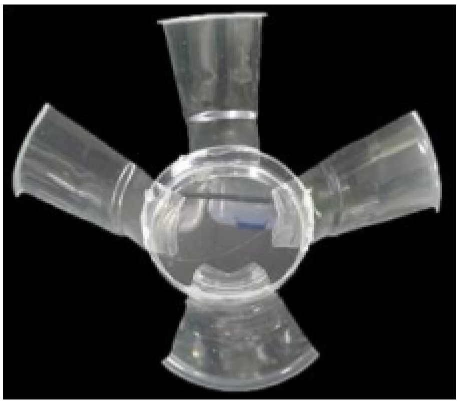

Figure S1. A home-made tray assembling the natural position of a maize leaf.

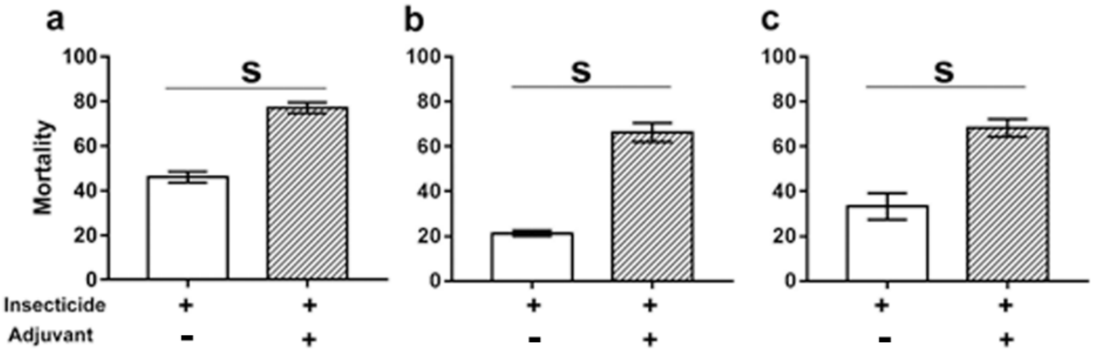

Figure S2. Mortality of FAW treated with insecticide with or without adjuvant Jijian®. The tested insecticides were 10 mg/L diflubenzuron (a), 10 mg/L pleocidin (b), and 100 mg/L beta-cypermethrin (c). Data are expressed as mean ± SE of at least three independent assays. s, significant difference by *t* test ($P<0.05$). +, insecticide or adjuvant present; −, insecticide or adjuvant absent.

Table S1. Efficacy evaluation of insecticides and their mixture with and without adjuvant in a field trial.

Treatment	Treatment details*	Trial plot code	Insect count				Inhibition of emergence (EI, %)			EI mean (%)			95% confidential limit of EI #					
			Pre-spray	Post-spray									day 1		day 3		day 7	
			day 0	day 1	day 3	day 7	day 1	day 3	day 7	day 1	day 3	day 7	95% CL of EI	Sig.	95% CL of EI	Sig.	95% CL of EI	Sig.
5% EB	15 g/ha.	1-1	25	5	0	1	73.82	100.00	88.52	80.79	89.96	91.24	75.24~86.34	cd	78.16~101.75	b	85.31~97.16	b
		1-2	29	4	3	1	81.95	83.85	90.10									
		1-3	21	2	0	1	87.54	100.00	86.33									
		1-4	26	4	4	0	79.86	75.98	100.00									
200g/L CT	150 ml/ha.	2-1	19	10	2	0	31.11	83.56	100.00	47.15	81.36	87.51	29.03~65.27	b	75.76~86.96	b	75.83~99.18	b
		2-2	20	4	3	2	73.82	76.58	71.29									
		2-3	27	12	4	1	41.83	76.87	89.37									
		2-4	27	12	2	1	41.83	88.43	89.37									
EB×CT I	13.5 g/ha. 5% EB + 0.375 mL/ha. 200g/L CT	3-1	14	3	2	1	71.95	77.69	79.49	70.80	86.50	89.35	65.32~76.29	bc	78.76~94.24	b	77.29~101.42	b
		3-2	26	5	3	2	74.83	81.98	77.92									
		3-3	21	6	1	0	62.61	92.56	100.00									
		3-4	25	5	1	0	73.82	93.75	100.00									
EB×CT II	2.7 g/ha. 5% EB + 0.075 mL/ha. 200g/L CT	4-1	19	6	7	3	58.67	42.47	54.67	58.26	68.83	76.47	47.17~69.34	bc	51.42~86.25	b	61.44~91.49	b
		4-2	20	8	3	1	47.65	76.58	85.65									
		4-3	25	5	4	1	73.82	75.02	88.52									
		4-4	25	9	3	2	52.88	81.26	77.03									
EB×CT II×adjuvant	2.7 g/ha. 5% EB +0.075 mL/ha. 200g/L CT +450 mL/ha. Adjuvant	5-1	22	6	3	2	64.30	78.71	73.90	71.71	86.98	84.37	60.74~82.68	c	80.30~93.65	b	73.09~95.64	b
		5-2	17	3	1	0	76.90	90.82	100.00									
		5-3	20	6	2	1	60.74	84.39	85.65									
		5-4	26	3	1	2	84.90	93.99	77.92									

Water	450 L/ha. tap water	6-1	25	20	18	8	-4.71	-12.42	8.13	-0.04	-0.05	-0.49	-3.67~3.59	a	-9.26~9.16	a	-7.53~6.56	a
		6-2	17	13	11	6	-0.09	-1.03	-1.33									
		6-3	26	19	15	9	4.36	9.92	0.62									
		6-4	21	16	13	8	0.28	3.34	-9.37									

*, ha., hectare

#, sig., significance among treatments were examine via ordinary one-way ANOVA. Different letters represent significant defferences.
